# Alveolar Epithelial Cells Promote IGF-1 Production by Alveolar Macrophages Through TGF-β to Suppress Endogenous Inflammatory Signals

**DOI:** 10.3389/fimmu.2020.01585

**Published:** 2020-07-21

**Authors:** Mimi Mu, Peiyu Gao, Qian Yang, Jing He, Fengjiao Wu, Xue Han, Shujun Guo, Zhongqing Qian, Chuanwang Song

**Affiliations:** ^1^Department of Immunology, School of Laboratory Medicine, Bengbu Medical College, Bengbu, China; ^2^Anhui Provincial Key Laboratory of Infection and Immunity, Bengbu Medical College, Bengbu, China; ^3^Anhui Province Key Laboratory of Immunology in Chronic Diseases, Bengbu Medical College, Bengbu, China

**Keywords:** AEC, AMs, IGF-1, TGF-β, inflammation

## Abstract

To maintain alveolar gas exchange, the alveolar surface has to limit unnecessary inflammatory responses. This involves crosstalk between alveolar epithelial cells (AECs) and alveolar macrophages (AMs) in response to damaging factors. We recently showed that insulin-like growth factor (IGF)-1 regulates the phagocytosis of AECs. AMs secrete IGF-1 into the bronchoalveolar lavage fluid (BALF) in response to inflammatory stimuli. However, whether AECs regulate the production of IGF-1 by AMs in response to inflammatory signals remains unclear, as well as the role of IGF-1 in controlling the alveolar balance in the crosstalk between AMs and AECs under inflammatory conditions. In this study, we demonstrated that IGF-1 was upregulated in BALF and lung tissues of acute lung injury (ALI) mice, and that the increased IGF-1 was mainly derived from AMs. *In vitro* experiments showed that the production and secretion of IGF-1 by AMs as well as the expression of TGF-β were increased in LPS-stimulated AEC-conditioned medium (AEC-CM). Pharmacological blocking of TGF-β in AECs and addition of TGF-β neutralizing antibody to AEC-CM suggested that this AEC-derived cytokine mediates the increased production and secretion of IGF-1 from AMs. Blocking TGF-β synthesis or treatment with TGF-β neutralizing antibody attenuated the increase of IGF-1 in BALF in ALI mice. TGF-β induced the production of IGF-1 by AMs through the PI3K/Akt signaling pathway. IGF-1 prevented LPS-induced p38 MAPK activation and the expression of the inflammatory factors MCP-1, TNF-α, and IL-1β in AECs. However, IGF-1 upregulated PPARγ to increase the phagocytosis of apoptotic cells by AECs. Intratracheal instillation of IGF-1 decreased the number of polymorphonuclear neutrophils in BALF of ALI model mice, reduced alveolar congestion and edema, and suppressed inflammatory cell infiltration in lung tissues. These results elucidated a mechanism by which AECs used TGF-β to regulate IGF-1 production from AMs to attenuate endogenous inflammatory signals during alveolar inflammation.

## Introduction

Continuous and direct exposure of tissues to the outside environment require tight regulation of the alveolar immune response to effectively identify and eliminate invading microorganisms with minimal immunopathology to maintain optimal gas exchange (1). Alveolar macrophages (AMs) constitute the first line of defense in the respiratory tract and are key regulators of airway immune responses. AMs account for 95% of lower respiratory tract leukocytes in the stable state (2). AMs actively inhibit antigen-induced T cell proliferation and decrease dendritic cell (DC) antigen presentation in the lungs (3). These immunosuppressive properties as well as the capacity to suppress airway hyper responsiveness confer AMs the ability to control the immune response and help preserve the physiological functions of the lungs.

The mature alveolar epithelium is composed of type I and type II alveolar epithelial cells (AECs or alveolar parietal cells), which occupy 95 and 5% of the alveolar surface, respectively (4). Under normal conditions, AECs are a slow proliferating lung cell population with a renewal time of 4–6 weeks. Unlike type I AECs, type II AECs can re-enter the cell cycle and divide. Therefore, type II AECs are considered pluripotent cells that possess high plasticity, self-renewal ability, and the capacity to produce type I AECs through the process of transdifferentiation (5). In addition to acting as a physical barrier against pathogens and various inhaled environmental particles, AECs actively participate in the lung's immune response, which is the main regulator of alveolar balance (6).

AMs are attached to the surface of AECs, supporting the possible interaction between the two cells (7). Therefore, many studies have examined the role of AM-AEC association and communication in lung homeostasis and inflammation. AEC-conditioned medium (AEC-CM) is chemotactic for AM migration; however, RANTES, MCP-1, and GM-CSF neutralizing antibodies decrease the chemotactic activity of AEC-CM for AMs by 58, 29, and 47%, respectively (8). AM-mediated suppression of cysteinyl leukotriene pathway genes in the AEC promotes resistance to influenza A virus infection, thereby protecting the body from fatal infection (9). Bourdonnay et al. showed that AMs secrete SOCS1 and 3 in exosomes and vesicles, which are ingested by AECs and inhibit the activation of JAK/STAT inflammatory signals in AECs (10).

Insulin-like growth factor (IGF)-1 is a 7.5 KDa peptide composed of 70 amino acids and has 50% homology to insulin. IGF-1 acts as an important survival factor in different cells by preventing apoptosis and inducing cell proliferation. IGF-1 plays an important role in the development and balance of organs and disease states, including the lung (11). Low serum IGF-1 level is a high risk factor for bronchoalveolar dysplasia (12). Pulmonary fibrosis is associated with increased IGF-1 originated from AMs, and IGF-1 can stimulate the differentiation of fibroblasts into myofibroblast phenotypes, thus playing an important role in pulmonary fibrosis (13–15). Narasaraju et al. showed that IGF-1 promotes the proliferation and differentiation of AECs (16). Recent work from our group showed that IGF-1 inhibits inflammatory signaling in AECs (17). However, whether IGF-1 acts as a mediator between AMs and AECs and its role in regulating the balance of the alveolar microenvironment remain unclear.

In the present study, we showed that TGF-β produced by LPS-induced AECs stimulated the production of IGF-1 by AMs. This AM-derived IGF-1 attenuated pre-inflammatory signals in AECs. This two-way communication between AECs and AMs represents a novel means for fine tuning inflammatory responses in alveoli.

## Materials and Methods

### AEC Cell Culture

The mouse AEC line MLE-12 was suspended in high glucose DMEM medium containing 10% fetal bovine serum (FBS), inoculated into 24-well plates, and cultured in a 37°C, 5% CO_2_ incubator. After the cells reached 70–90% confluency, they were digested with 0.25% trypsin, collected, and passaged every other day.

### Antibodies and Reagents

LPS (Escherichia coli O26:B6, L8274) was obtained from Sigma-Aldrich. TGF-β1 (CK33) was purchased from novoprotein company. TGF-β1 antibody (sc-146) was purchased from santa cruz biotechnology company. IGF-1 (MB-2917A) was purchased from Jiangsu Enzyme Biotech company. IGF-1 antibody (ab9572) was originated from abcam company. PPARγ antibody (16643-1-AP) was purchased from Proteintech. p-AKT antibody (AA329-1), phosphorylated P38MAPK antibody (AM063-1), GAPDH antibody (AG019-1), HRP-labeled goat anti-mouse antibody (A0216), HRP-labeled goat anti-rabbit antibody (A0208), PI3K inhibitor Wortmannin (S1952), Annexin V-FITC dye (C1062L), and TGF-β isotype antibody (A7007) were from Beyotime. Curcumin (458-37-7) was purchased from meilunbio company. Pirfenidone (53179-13-8) was from MedChemExpress. Lipo fectamine2000 (1854311) was purchased from Invitrogen.

### Establishment of the Acute Lung Injury Model

BALB/c mice were randomly divided into a normal control group and an acute lung injury (ALI) group. ALI model mice were challenged with lipopolysaccharide (LPS, 4 mg/kg) through a single nasal instillation, and the normal control group was administered PBS instead of LPS. Mice were sacrificed 24 h after LPS challenge, and the bronchoalveolar lavage fluid (BALF) and lung tissues were collected and stored at −80°C until use.

### BAL and Acquisition of BALF

Under anesthesia, the mice were intubated, and bronchoalveolar lavage was performed using 1 ml of sterile PBS six times. The BALF was centrifuged at 1,500 rpm for 5 min, and the supernatant was collected and stored at −80°C until use. Cells in BALF were washed twice with PBS, and part of the cell suspension was used for cell counting, whereas the other part was subjected to cell smear. Wright staining was performed, and the cells were classified and counted under a microscope.

### Acquisition of Primary AMs

The BALF obtained as described above was centrifuged and resuspended in RPMI-1640 complete medium. The cell suspension was inoculated into a 6-well plate (1 × 10^6^ cells/well) and cultured in a 37°C incubator for 2 h. The suspended cells were aspirated, and the adherent cells were kept as the AM fraction.

### Induction and Labeling of Apoptotic Cells

The mouse alveolar epithelial cell line MLE-12 was resuspended in DMEM complete medium and seeded into 6-well plates (1 × 10^6^ cells/well). After the cells became adherent, they were stimulated with 50 μM curcumin for 72 h. Cells were then collected and stained for 15 min with AnnexinV-FITC in the dark. The stained apoptotic cells were washed twice with cold sterile PBS for use.

### Detection of Phagocytosis

MLE-12 cells were resuspended in DMEM complete medium and seeded into 6-well plates (10^5^ cells/well). After adherence of the cells, FITC-labeled apoptotic cells (5 × 10^4^ cells/well) were added and co-cultured for 4 h. The cells were collected, washed twice with PBS, and the cell suspension was fixed with 4% PFA. Flow cytometry was used to detect the phagocytosis of FITC-labeled apoptotic cells.

### RNA Interference

MLE-12 cells (10^5^ cells/well) were seeded into 6-well plates and allowed to adhere for 12 h before transfection. Cells were transfected using Lipofectamine® 2000 following the manufacturer's instructions. A 80 nM FAM-siRNA-Lipo2000 mixture was prepared according to the instructions. MLE-12 cells were co-cultured with this mixture in 400 μL FBS-free DMEM for 48 h, and protein was extracted from cells to verify the transfection efficacy.

### The qRT-PCR

Total RNA was extracted using the TRIzol reagent. The first strand cDNA was synthesized using 2 μg of RNA as a template according to the kit instructions (RT TransScript First-Strand cDNA Synthesis SuperMix kit). Then, primers were designed and real-time PCR was performed according to the kit instructions (SYBRGreen TransStart Top Green qPCR SuperMix kit). The relative expression of each gene was analyzed using the 2^−ΔΔ*Ct*^ method. The primer sequences (5′-3′) are listed below: PPARγ, Sense: ACTCATACATAAAGTCCTTCCCGC, Antisense: CTCTTGCACGGCTTCTACGG; LXRA, Sense: TCATCAAGGGAGCACGCTATGT, Antisense: CTTGAGCCTGTTCCTCTTCTTGC; LXRB, Sense: TCCGACCAGCCCAAAGTCAC, Antisense: GCTGTTTCTAGCAACATGATCTCAA; TNF-α, Sense: ACCCTCACACTCACAAACCA, Antisense: ATAGCAAATCGGCTGACGGT; IL-1β, Sense: AAAAGCCTCGTGCTGTCG, Antisense: TGCTTGTGAGGTGCTGATGTA; MCP-1, Sense: GTCCCTGTCATGCTTCTGG, Antisense: AAGTGCTTGAGGTGGTTGTG; GAPDH, Sense: CCTCGTCCCGTAGACAAAATG, Antisense: TGAGGTCAATGAAGGGGTCGT.

### Western Blot Analysis

Protein was extracted from cells using NP-40 solution, and protein concentration was determined using the BAC method. Aliquots containing 30 μg of protein were separated by 6% SDS-polyacrylamide gel electrophoresis, followed by transfer to a nitrocellulose membrane. The membrane was blocked with 5% milk for 2 h, and then incubated with the following primary antibodies at 4°C overnight: IGF-1 (1: 500), p-Akt (1: 1000), PPARγ (1: 1000), p-p38 MAPK (1: 2000), and GAPDH (1: 1000). The membrane was washed with Tris-buffered saline containing 0.05% Tween-20 and incubated with HRP-labeled goat anti-mouse antibody (1: 2000) for 2 h. Bands on the membrane were visualized using a BeyoECL Plus kit and integrated optical density analysis was performed using Image J software.

### Wet-Dry Weight Ratio of Lung Tissue

The lung tissues of mice in each group were collected, and PBS pulmonary arterial lavage was performed to remove residual blood. Lung tissues were placed on absorbent paper to eliminate surface moisture, and the weight (wet weight) was measured uniformly and recorded. Lung tissues were then placed in a 37°C incubator for 24 h until the weight became constant. Then, lung tissues were removed and weighed (dry weight). The wet/dry (W/D) weight ratio of lung tissues in each group of mice was calculated.

### Determination of Protein Concentration in BALF

Mice were tracheally intubated, and the BALF was obtained as described above. The protein concentration in BALF was measured according to the kit instructions.

### HE Staining of Lung Tissue

Mouse lung tissues were fixed for 24 h with 4% paraformaldehyde and then dehydrated for 12 h using a fully automatic tissue dehydrator. Lung tissues were embedded in paraffin, and paraffin blocks were cut into 5 μm thick slices on a microtome. The sections were dewaxed with different concentrations of xylene, and after immersion in a gradient of alcohol (high concentration to low concentration), tissues were stained with hematoxylin and eosin. The sections were transparent with xylene and then sealed with a neutral resin, and observed and photographed under a microscope.

### Statistical Methods

Experimental data are expressed as the mean ± standard deviation. Data were analyzed using SPSS 16.0 software. Comparisons between multiple groups were performed using analysis of variance, and comparisons between two groups were performed using *t*-tests. *P* < 0.05 was considered statistically significant.

## Results

### Increased IGF-1 Production in Acute LPS Lung Injury Models

**Figure 1:**
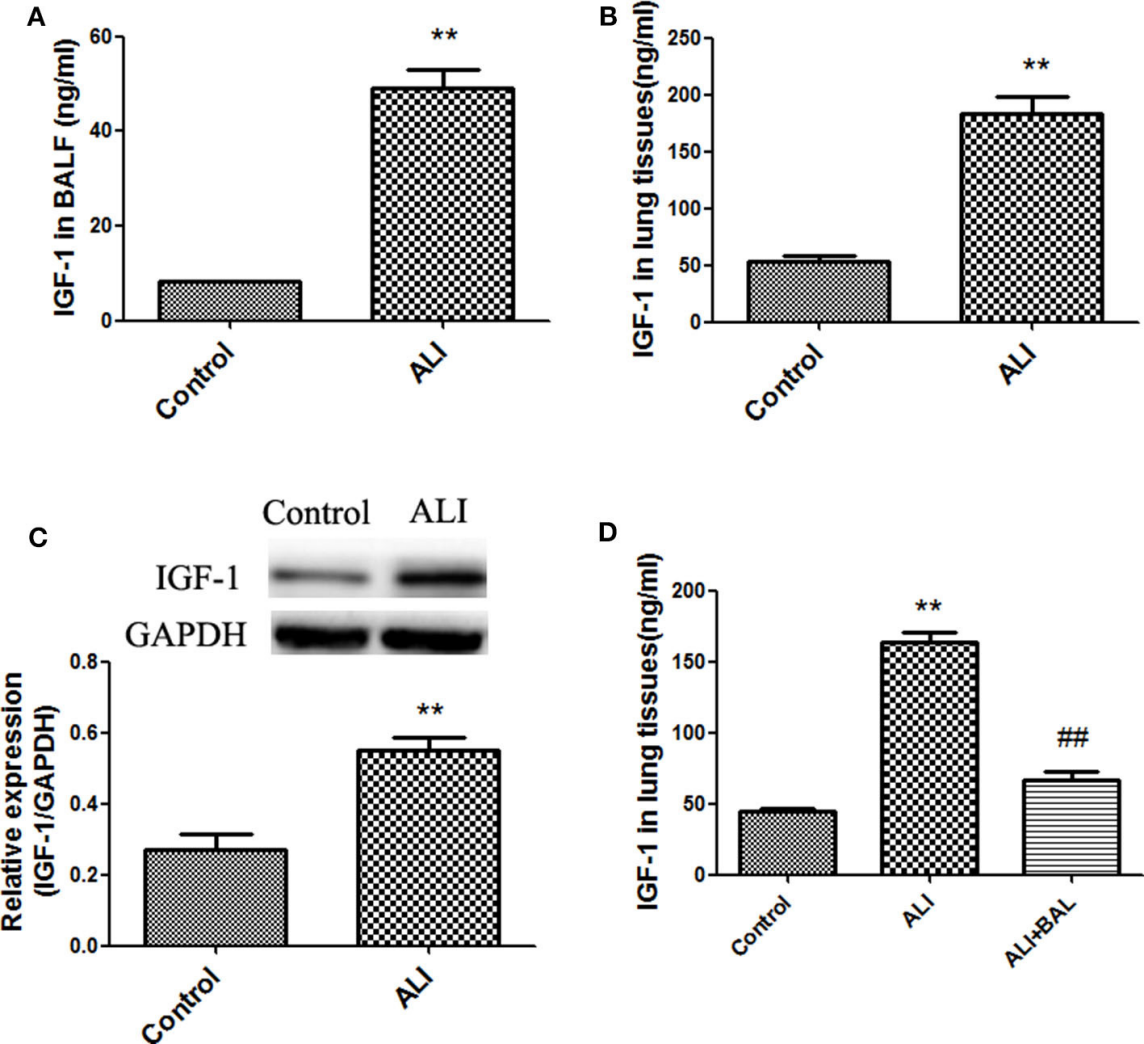
Increased IGF-1 production in acute LPS lung injury models. **(A–D)** BALB/c mice were instilled with LPS (4 mg/kg) into the nasal cavity to develop an ALI model. After 24 h of LPS instillation, IGF-1 content was detected in BALF **(A)** and lung tissue homogenates **(B)**, and the expression of the IGF-1 protein in lung tissues **(C)** was detected by immunoblotting. **P < 0.01 vs. control group. **(D)** After ALI model mice were subjected to BAL (ALI + BAL), lung tissues were collected for homogenization, and the IGF-1 content in the homogenate was detected by ELISA. **P < 0.01 vs. the control group, ^*##*^*P* < 0.01 vs. the ALI group.

Recent studies show that IGF-1 is involved in the regulation of inflammation (18). We first examined the expression and secretion of IGF-1 in the lungs of mice with LPS-induced ALI. In these experiments, IGF-1 was quantitatively detected by ELISA in BALF and lung tissue homogenates. At 24 h after LPS administration, the content of IGF-1 was significantly higher in the BALF and lung tissue homogenates of treated mice than in those of control mice (Figures 1A,B), and the expression of IGF-1 in lung tissues was also increased (Figure 1C). The increased IGF-1 content in the lung tissue homogenate was reduced by 60% after BAL in LPS lung injury model mice (Figure 1D). This indicated that a large amount of IGF-1 in the lung tissue of ALI mice were removed by BAL.

### IGF-1 Is Derived From AMs in the ALI Model

**Figure 2:**
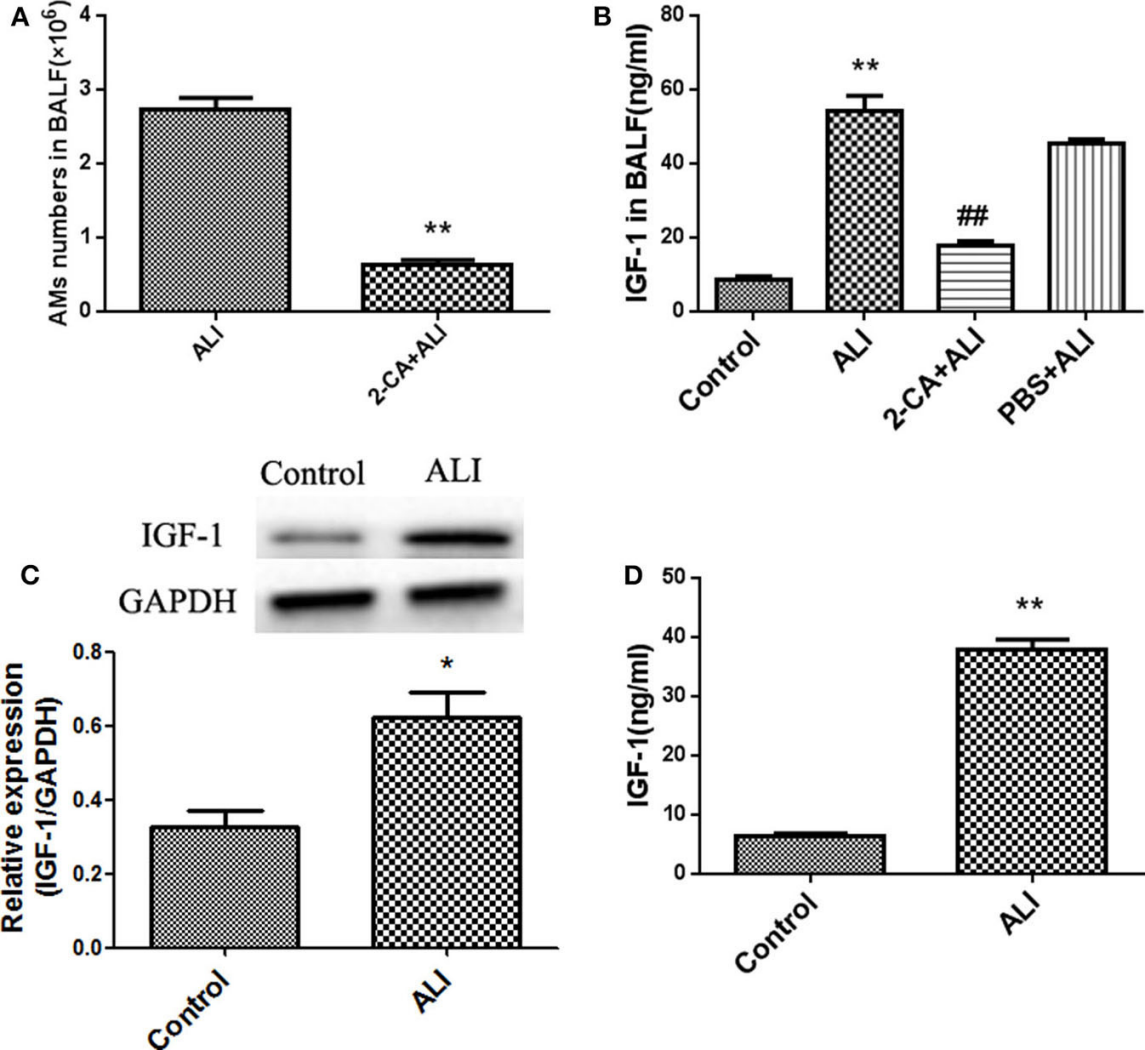
IGF-1 is derived from AMs in the ALI model. **(A–D)** ALI model mice were generated as described in Materials and Methods (Antibodies and Reagents). The 2-CA (10 μg 2-CA in 20 μL PBS) was dripped into the nasal cavity for 12 h before the generation of the ALI model, and AMs were removed from ALI model mice (2-CA + ALI). Mice in the PBS + ALI group were ALI model mice treated with PBS instead of 2-CA. **(A)** After 24 h of LPS instillation, primary AMs were obtained and counted as described in Materials and Methods (BAL and Acquisition of BALF). **P < 0.01 vs. the ALI group. **(B)** BALF was obtained as described in Materials and Methods (Establishment of the Acute Lung Injury Model), and the IGF-1 content was detected by ELISA. **P < 0.01 vs. the control group, ^*##*^*P* < 0.01 vs. the ALI group. **(C,D)** The expressions of AM band IGF-1 in normal control and ALI model mice were detected by immunoblotting **(C)** and ELISA **(D)**, respectively. *P < 0.05, **P < 0.01 vs. the control group.

IGF-1 is a polypeptide with a molecular weight of 7.6 KDa. Despite reports of IGF-1 produced after stimulation of epithelial cells and endothelial cells, studies show that the increased IGF-1 in the lungs of patients with allergic pneumonia is mainly derived from AMs (15). We therefore examined whether the elevated IGF-1 in the ALI model was derived from AMs. AMs were removed by 2-CA airway instillation, which showed that >77% of AMs were removed from ALI mouse airways (Figure 2A). After administration of 2-CA into the airway for 12 h, LPS was administered to generate the ALI model. The IGF-1 content in the BALF was significantly lower in 2-CA + ALI mice than in the LPS alone group (Figure 2B). In addition, the expression and secretion of IGF-1 in primary AMs of ALI model mice were significantly higher than those of normal control mice (Figures 2C,D). These results indicate that IGF-1 in the airway is mainly produced by AMs in mice exposed to ALI.

### IGF-1 Production Is Enhanced in AMs by LPS-Stimulated AEC-CM

**Figure 3:**
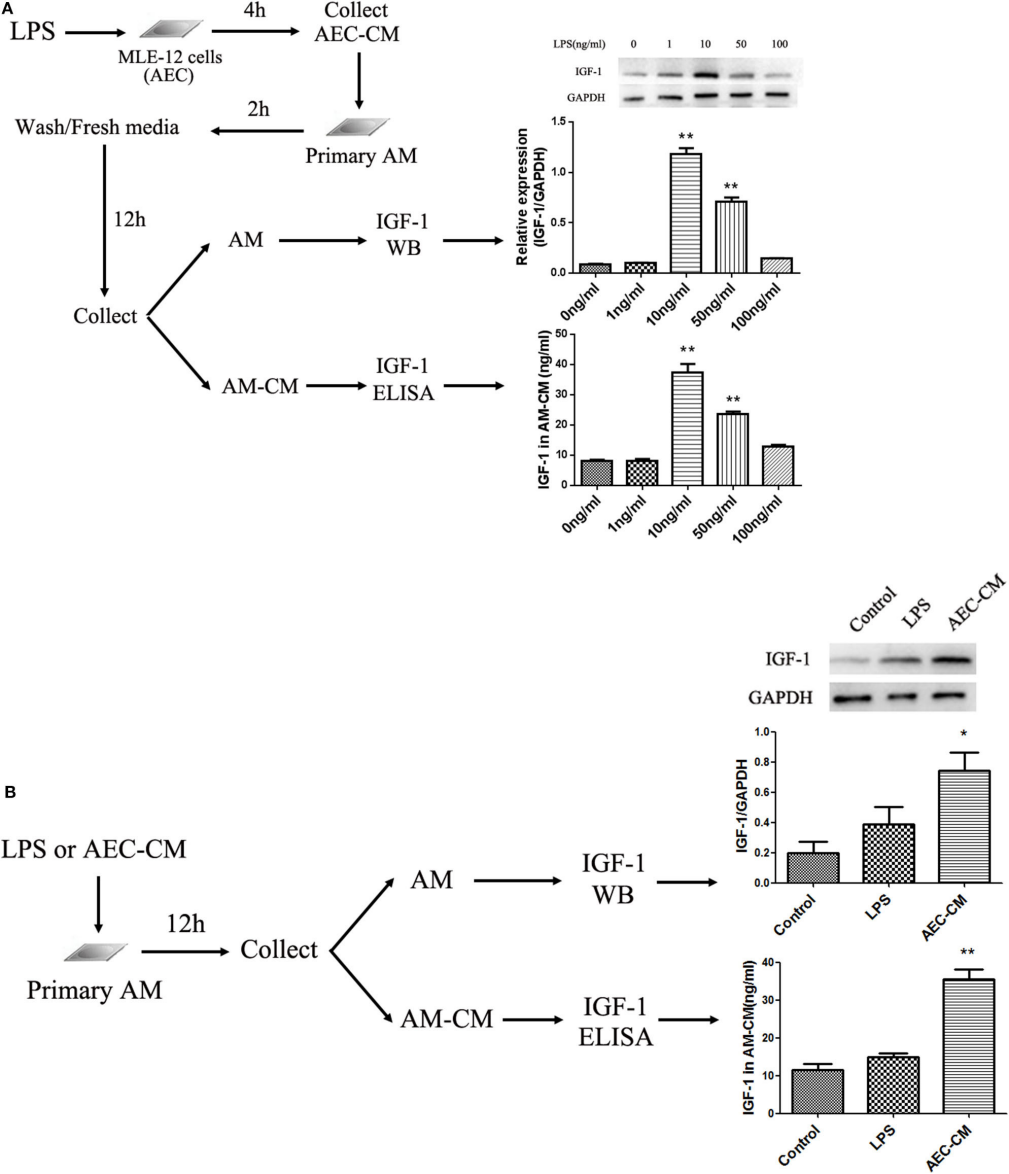
IGF-1 production is enhanced in AMs by LPS-stimulated AEC-CM. **(A)** AECs (1 ×10^6^ cells/well) were inoculated into a 6-well plate and allowed to adhere before treatment with different doses of LPS (1, 10, 50, and 100 ng/mL) for 4 h and collection of AEC-CM. Primary AMs were extracted as described in Materials and Methods and cultured (1 ×10^6^ cells/well). After the cells adhered to the wall, AEC-CM was added for 2 h. After several washes, AMs were resuspended in RPMI-1640 complete medium and cultured for an additional 12 h. AM-CM was collected and the IGF-1 content was detected by ELISA. Immunoblotting was used to detect IGF-1 protein expression in AMs. **P < 0.01 vs. the 0 ng/mL group. **(B)** Primary AMs (1 ×10^6^ cells/well) were inoculated into a 6-well plate and stimulated with LPS (10 ng/mL) or AEC-CM (10 ng/mL, LPS stimulation group) for 12 h. The expression of AM IGF-1 was detected by immunoblotting, and the content of IGF-1 in AM-CM was detected by ELISA. *P < 0.05, **P < 0.01 vs. the control group.

LPS induced IGF-1 production *in vivo*, and airway IGF-1 was mainly produced by AMs in ALI. This suggested that IGF-1 was produced by LPS-stimulated AMs or alternatively, a factor derived from LPS-stimulated AECs induces AMs to produce IGF-1. To test these possibilities, we examined the indirect effects of LPS-activated AEC-derived secretion factors and the direct effects of LPS on IGF-1 secretion by AMs. Mouse-derived MLE-12 AECs were treated with different doses of LPS for 4 h, and the AEC-CM produced was added to mouse primary AMs for 2 h. IGF-1 production was measured by western blotting and ELISA (Figure 3A). LPS increased the ability of AMs to secrete and express IGF-1 in the AEC-CM in a dose-dependent manner, and the greatest effect was observed at a dose of 10 ng/mL LPS. Direct treatment of primary AMs with LPS for 12 h also increased the production of IGF-1, although the increase did not reach statistical significance (Figure 3B). These results indicated that the production of IGF-1 by AMs was induced by a factor secreted by LPS-stimulated AECs. Therefore, in subsequent experiments, we focused on identifying the AEC-derived factor responsible for this effect.

### Identification of Factors Produced by LPS-Treated AECs That Induce IGF-1 Secretion by AMs

**Figure 4:**
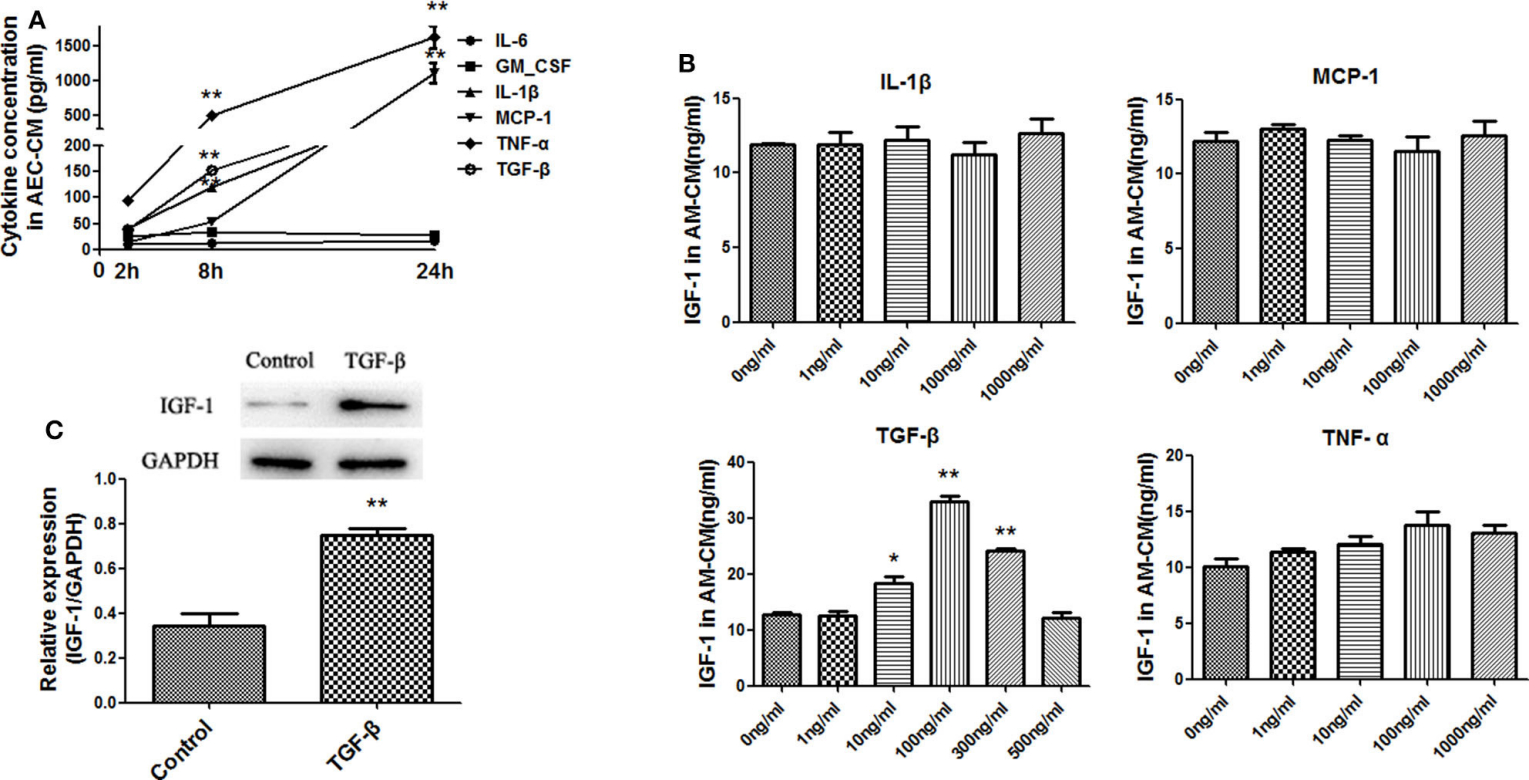
Identification of factors produced by LPS-treated AECs that induce IGF-1 secretion by AMs. **(A)** AECs (2 ×10^5^ cells/well) were inoculated into a 24-well plate and stimulated with LPS (10 ng/mL) for 2, 8, and 24 h. The supernatants were analyzed by ELISA for IL-6, GM-CSF, IL-1β, MCP- 1, TNF-α, and TGF-β content. **P < 0.01 vs. the 2 h group. **(B)** Primary AMs (1 ×10^6^ cells/well) were inoculated into 6-well plates and stimulated h with different concentrations (1, 10, 100, and 1,000 ng/mL) of IL-1β, TNF-α, MCP-1, and TGF-β for 24 h. The IGF-1 content in AM-CM was measured by ELISA. *P < 0.05 and **P < 0.01 vs. the 0 ng/mL group. **(C)** Primary AMs (1 ×10^6^ cells/well) were inoculated into 6-well plates and stimulated with TGF-β (100 ng/mL) for 24 h. The expression level of IGF-1 in AMs was detected by western blotting. **P < 0.01 vs. the control group.

AECs secrete a variety of mediators to regulate AM activity. Among them, MCP-1, IL-6, TNF-α, IL-1β, GM-CSF, and TGF-β are induced by LPS (19–22). Therefore, the levels of these factors in AECs stimulated with LPS (10 ng/mL) for 2, 8, and 24 h were measured by ELISA to determine the effect of LPS in our experimental system. As shown in Figure 4A, LPS strongly upregulated TNF-α and moderately upregulated MCP-1, IL-1β, and TGF-β, whereas IL-6 and GM-CSF did not increase significantly. To determine whether these factors could induce IGF-1 secretion from AMs, primary AMs were directly exposed to each mediator for 1 h *in vitro*, and the IGF-1 content in the AM-CM produced was analyzed by ELISA. As shown in Figure 4B, TGF-β was the only factor that significantly increased IGF-1 levels in AM-CM. In addition, TGF-β stimulation upregulated the expression of IGF-1 in AMs (Figure 4C). These results suggested that TGF-β was a likely AEC-derived candidate factor inducing IGF-1 secretion by AMs in the context of LPS stimulation.

### TGF-β Is Necessary for IGF-1 Production in AMs Induced by LPS-Stimulated AEC-CM

**Figure 5:**
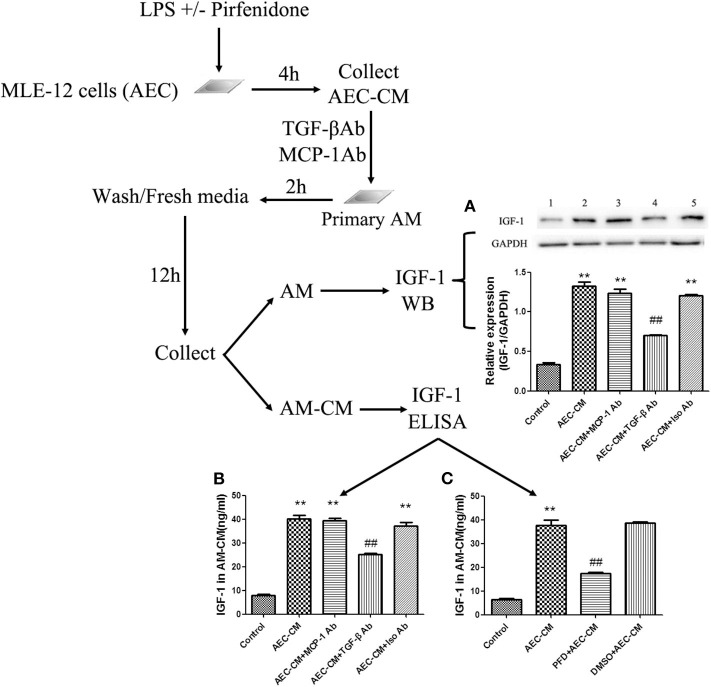
TGF-β is necessary for IGF-1 production in AMs induced by LPS-stimulated AEC-CM. **(A–C)** AECs (1 ×10^6^ cells/well) were inoculated into a 6-well plate, stimulated with LPS (10 ng/mL) for 4 h, and AEC-CM was collected. AEC-CM was incubated for 30 min at 4°C with anti-MCP-1 antibody (MCP-Ab) or anti-TGF-β antibody (TGF-β Ab) or isotype control antibody (Iso Ab). Primary AMs were stimulated with the treated AEC-CM, washed, and resuspended in RPMI-1640 complete medium for 12 h. The content of IGF-1 in AM-CM was detected by ELISA **(A)**, and the expression of IGF-1 in AM was detected by western blotting **(B)**. In addition, AECs were treated with pirfenidone (50 ng/μL) for 1 h before stimulation with LPS (10 ng/mL), and the AEC-CM was collected and added to AMs for 2 h. After washing and resuspending AMs in culture medium for 12 h, the IGF-1 content in AM-CM was detected by ELISA **(C)**. **P < 0.01 vs. the control group, ^*##*^*P* < 0.01 vs. the AEC-CM group.

Antibody neutralization experiments were used to assess the effect of AEC-derived TGF-β on AMs. Addition of a neutralizing antibody against TGF-β to AEC-CM blocked the ability of AEC-CM to induce the expression and secretion of IGF-1 by AMs, whereas a neutralizing antibody against MCP-1 had no effect (Figure 5A). To confirm these results, AECs were treated with a TGF-β synthesis inhibitor before LPS stimulation to block TGF-β production from AECs. This treatment partially blocked the ability of AEC-CM to induce IGF-1 secretion from AMs (Figures 5B,C). Taken together, these results suggested that TGF-β was an important mediator originated from LPS-stimulated AECs that induces IGF-1 secretion from AMs.

### IGF-1 Elevation in the ALI Model Is TGF-β Dependent

**Figure 6:**
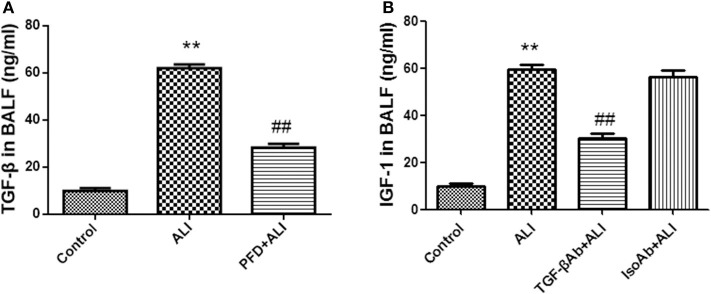
IGF-1 elevation in the ALI model is TGF-β dependent. **(A,B)** ALI model mice were generated as described in Materials and Methods (Antibodies and Reagents). At 24 h before LPS instillation, pirfenidone (400 μg/kg) **(A)** or TGF-β antibody (400 μg/kg) **(B)** was instilled into the nasal cavity once every 12 h. At 24 h after LPS instillation, the content of TGF-β in the BALF of each group of mice was measured by ELISA. **P < 0.01 vs. the control group, ^*##*^*P* < 0.01 vs. the ALI group.

Instillation of LPS into the airways of mice upregulated TGF-β in BALF (Figure 6A). To determine whether TGF-β was involved in the LPS-induced increase in IGF-1 production *in vivo*, mice were treated with the TGF-β synthesis inhibitor pirfenidone 24 h before LPS stimulation. Pirfenidone partially blocked the LPS-induced increase in IGF-1 in the lungs (Figure 6A). In addition, treatment with anti-TGF-β antibody by intratracheal instillation before LPS stimulation significantly reduced IGF-1 content in BALF in ALI model mice (Figure 6B). These results indicated that TGF-β acted as a mediator that promotes the secretion of IGF-1 by AMs in response to LPS stimulation.

### TGF-β Induces IGF-1 Production in AMs via the PI3K/Akt Signaling Pathway

**Figure 7:**
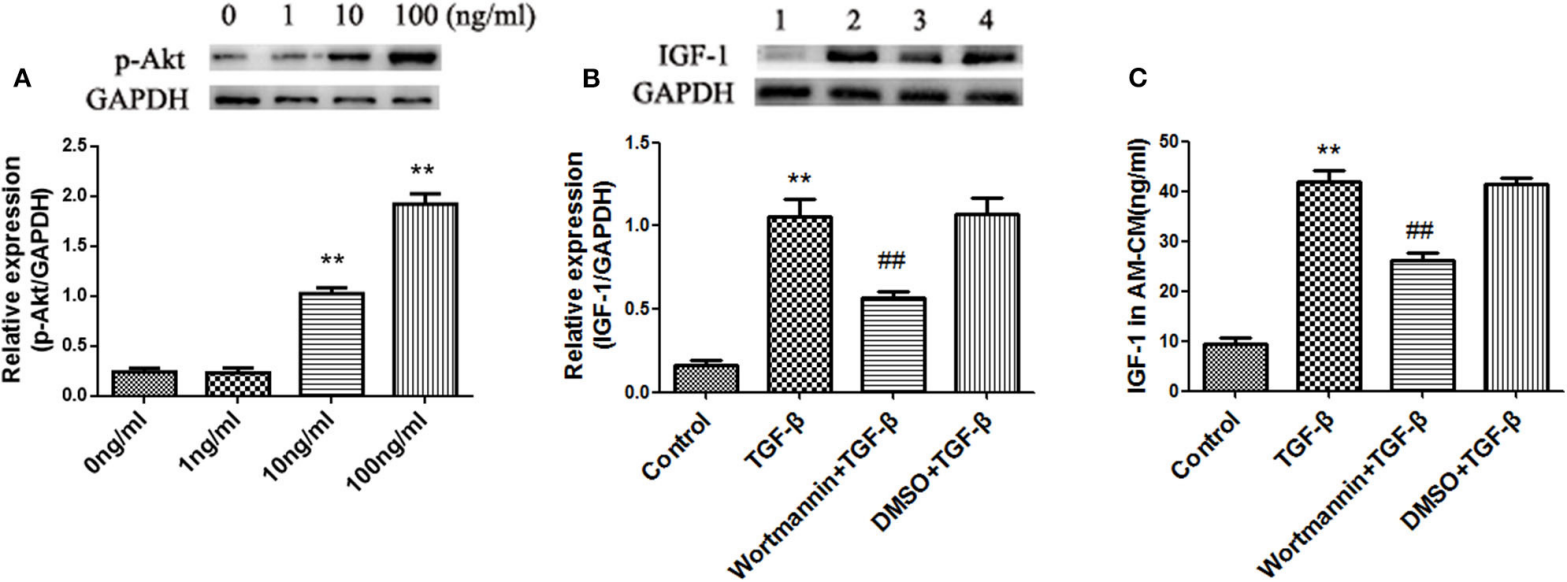
TGF-β induces IGF-1 production in AMs via the PI3K/Akt signaling pathway. **(A)** Primary AMs (1 ×10^6^ cells/well) were inoculated into 6-well plates and stimulated with different concentration of TGF-β (0, 1, 10, and 100 ng/mL) for 24 h. The expression of p-Akt in AMs was detected by western blotting. **P < 0.01 vs. the 0 ng/ml group. **(B,C)** Primary AMs (1 ×10^6^ cells/well) were inoculated into a 6-well plate and stimulated with Wortmannin (1 mM) for 2 h, followed by TGF-β (100 ng/mL) treatment for 24 h. IGF-1 protein expression in AMs was detected by immunoblotting, and the IGF-1 content in AM-CM was detected by ELISA. **P < 0.01 vs. the control group, ^*##*^*P* < 0.01 vs. the TGF-β group.

Not only does TGF-β activate Smad signaling, but also it can activate the PI3K/Akt signaling pathway (23). We showed that TGF-β activated Akt in AMs in a dose-dependent manner (Figure 7A), which suggested TGF-β activated the PI3K/Akt signaling pathway in AMs. Blocking the PI3K signaling pathway with Wortmannin partially suppressed the expression and secretion of IGF-1 in AMs induced by TGF-β (Figures 7B,C). These results indicated that TGF-β induced the production of IGF-1 in AMs through the PI3K/Akt signaling pathway.

### IGF-1 Prevents LPS-Induced p38 MAPK Activation and MCP-1, TNF-α, and IL-1β Production in AECs

**Figure 8:**
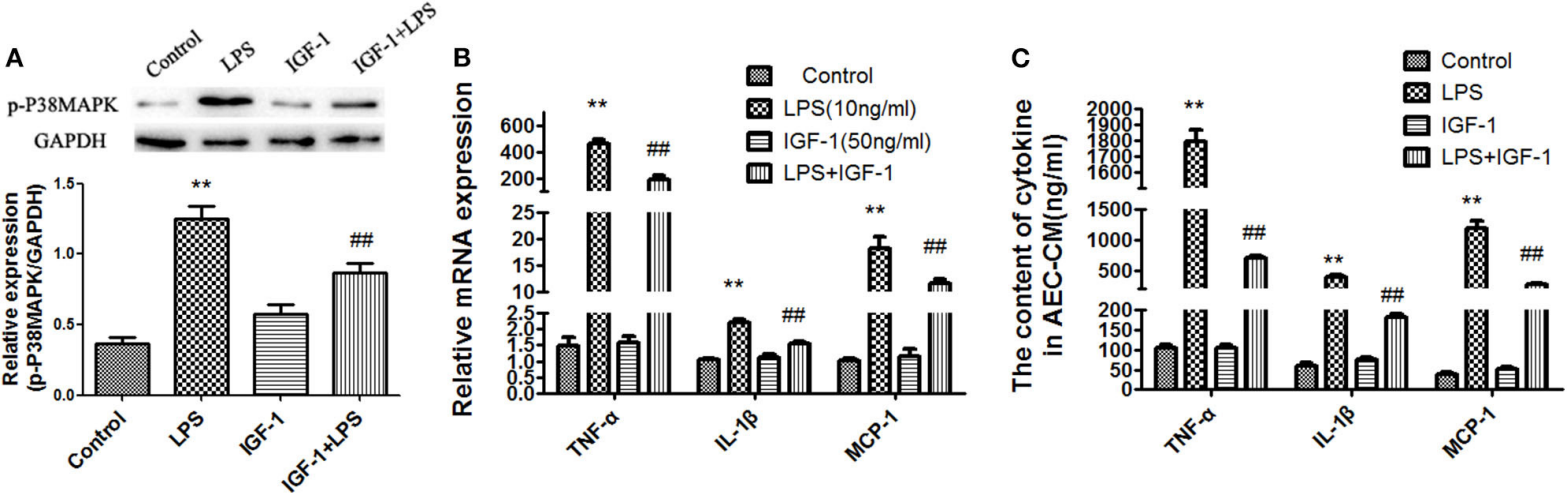
IGF-1 prevents LPS-induced p38 MAPK activation and MCP-1, TNF-α, and IL-1β production in AECs. **(A–C)** AECs (1 ×10^6^ cells/well) were inoculated into a 6-well plate and pre-stimulated with IGF-1 (50 ng/mL) for 1 h, followed by LPS (10 ng/mL) for a total of 24 h. **(A)** The expression of p-p38 MAPK in AECs was detected by immunoblotting. **(B)** The mRNA expression levels of the cytokines TNF-α, IL-1β, and MCP-1 in AECs was detected by qRT-PCR. **(C)** The cytokines TNF-α, IL-1β, and MCP-1 in AEC-CM were detected by ELISA. **P < 0.01 vs. the control group, ^*##*^*P* < 0.01 vs. the LPS group.

We showed that LPS-treated AECs induced IGF-1 secretion by AMs through the release of TGF-β. Therefore, we investigated the functional effects of IGF-1 on AEC inflammation-related signaling in AECs. For this purpose, AECs were pretreated with IGF-1 for 2 h and then stimulated with LPS before analysis of p38 MAPK activation and expression of inflammatory factors. Compared with the LPS single stimulation group, pretreatment with IGF-1 significantly prevented LPS-induced p38 MAPK activation (Figure 8A) and MCP-1, TNF-α, and IL-1β expression in AECs (Figure 8B). Similarly, before stimulation of AECs with LPS, pretreatment with IGF-1 for 1 h significantly reduced LPS-induced secretion of MCP-1, TNF-α, and IL-1β in AECs (Figure 8C).

### IGF-1 Promotes Apoptotic Cell Phagocytosis by AECs Through PPARγ

**Figure 9:**
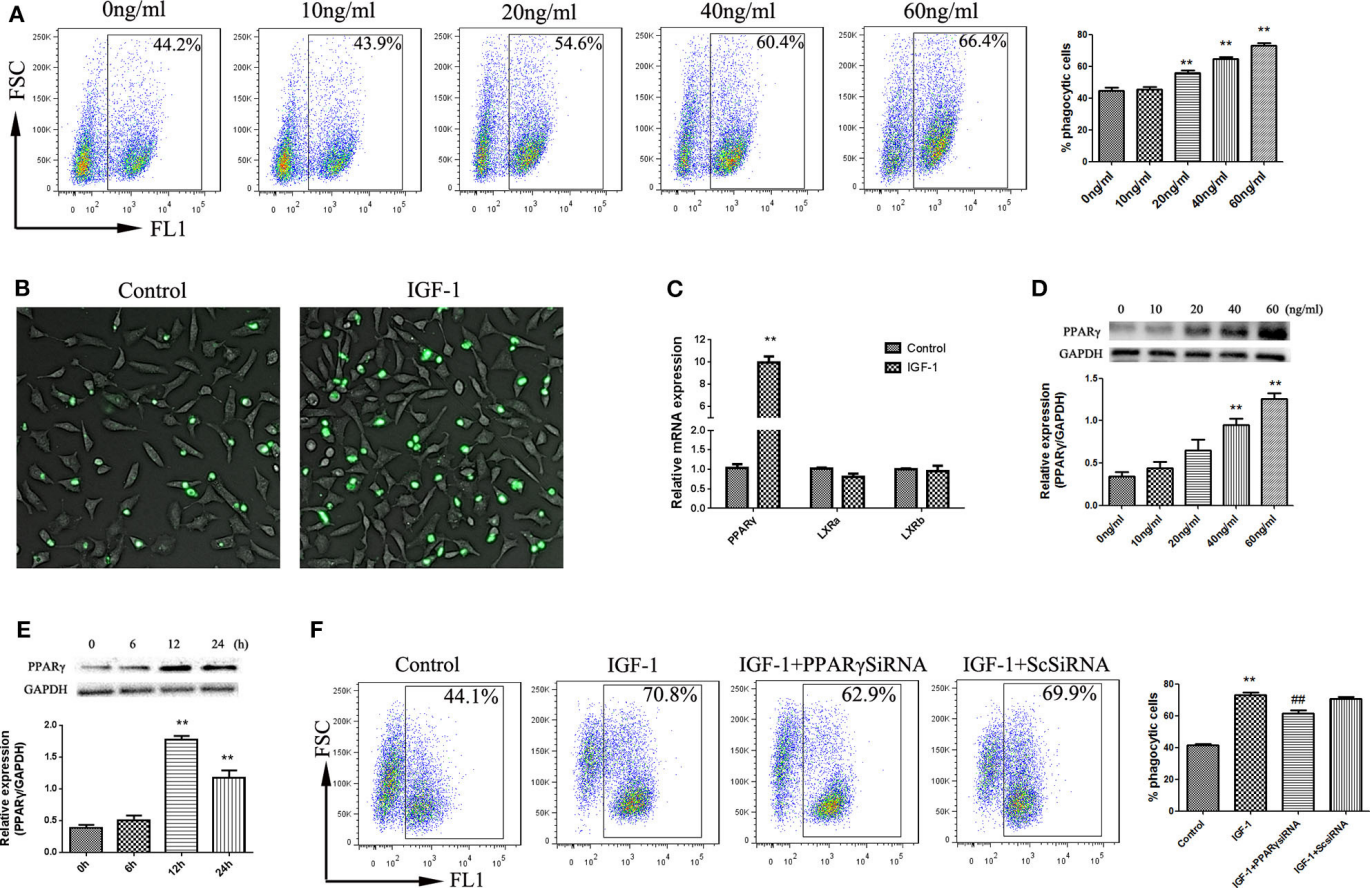
IGF-1 promotes apoptotic cell phagocytosis by AECs through PPARγ. **(A,B)** AECs (1 ×10^5^ cells/well) were inoculated into a 6-well plate, stimulated with different concentrations of IGF-1 (0, 10, 20, 40, and 60 ng/mL) for 12 h, and co-cultured with FITC-labeled apoptotic cells for a total of 4 h. Phagocytosis of apoptotic cells was detected by flow cytometry **(A)** and fluorescence microscopy **(B)**. **P < 0.01 vs. the 0 ng/mL group. **(C)** AECs (1 ×10^6^ cells/well) were inoculated into a 6-well plate, stimulated with IGF-1 (60 ng/mL) for 24 h, and qRT-PCR was used to detect PPARγ, LxRA, and LxRB mRNA expression in AECs. **P < 0.01 vs. the control group. **(D)** AECs (1 ×10^5^ cells/well) were inoculated into a 6-well plate and treated with IGF-1 at different concentrations (0, 10, 20, 40, and 60 ng/mL) for 24 h. The protein expression of PPARγ was detected by western blotting. **P < 0.01 vs. the 0 ng/ml group. **(E)** AECs were stimulated with IGF-1 (60 ng/mL) for 6, 12, and 24 h, and the protein expression of PPARγ in AECs was detected by western blotting. **P < 0.01 vs. the 0 h group. **(F)** AECs (1 ×10^5^ cells/well) were inoculated into a 6-well plate, transfected with PPARγ siRNA or scrambled (Sc) siRNA for 36 h, and stimulated with IGF-1 (60 ng/mL) for 12 h. FITC-labeled apoptotic cells were added to the culture for 4 h. AEC phagocytosis of apoptotic cells was detected by flow cytometry. **P < 0.01 vs. the control group, ^*##*^*P* < 0.01 vs. the IGF-1 group.

Phagocytosis is the process by which phagocytes take up particles larger than 0.5 μm in diameter. Phagocytosis of apoptotic cells plays an important role in the resolution of inflammation and the maintenance of tissue balance (24). We therefore examined the effect of IGF-1 on AEC phagocytosis for apoptotic cells. As shown in Figures 9A,B, IGF-1 promoted phagocytosis of apoptotic cells by AECs in a dose-dependent manner, and 50 ng/ml IGF-1 had the strongest effect. Because PPARγ and LXR are involved in phagocytosis, we investigated the role of these transcription factors in IGF-1-induced phagocytosis in AECs. IGF-1 had no effect on LXR expression in AECs (Figure 9C); therefore, we focused on PPARγ in subsequent experiments. IGF-1 upregulated PPARγ in AECs in a time- and dose-dependent manner (Figures 9D,E), indicating that IGF-1 signaling induced the expression of PPARγ in AECs. PPARγ gene interference inhibited the effect of IGF-1 on the phagocytosis of apoptotic cells by AECs (Figure 9F). This indicated that IGF-1 promoted the phagocytosis of apoptotic cells by AECs by inducing the expression of PPARγ.

### IGF-1 Reduces Airway Inflammation in ALI Mice

**Figure 10:**
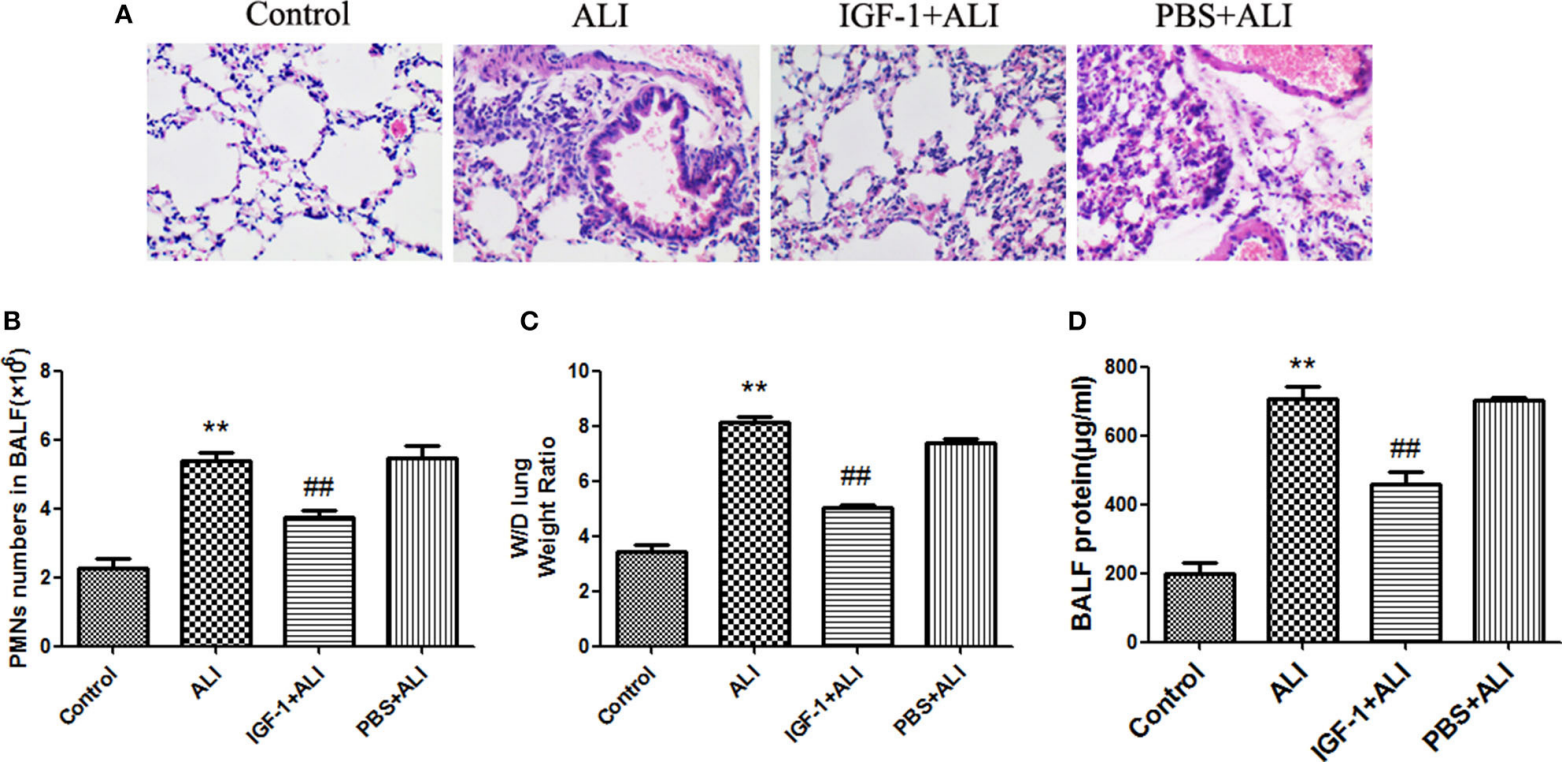
IGF-1 reduces airway inflammation in ALI mice. **(A–D)** ALI model mice were prepared as described in Materials and Methods (Antibodies and Reagents). At 24 h before LPS nasal drops, BALB/c mice received nasal drops of IGF-1 (0.8 mg/kg) once every 12 h. At 24 h after LPS treatment, lung tissues and BALF were collected from mice. **(A)** The lung tissues of each group were stained with HE, and the pathological damage was observed under a microscope. **(B)** The BALF of each group of mice was centrifuged, cell pellets were smeared and stained with Wright's staining solution, and the number of neutrophils was counted. **(C)** Wet-dry weight ratios of lung tissues in each group of mice were calculated as described in Materials and Methods (Western Blot Analysis). **(D)** The total protein content of BALF in each group of mice was measured according to Materials and Methods (Wet-Dry Weight Ratio of Lung Tissue). **P < 0.01 vs. the control group, ^*##*^*P* < 0.01 vs. the ALI group.

Elimination of apoptotic cells contributes to the resolution of inflammation. *In vitro* experiments demonstrated that IGF-1 promotes apoptotic cell phagocytosis by AECs and inhibits LPS-induced inflammatory signals. Therefore, we next investigated the potential effect of IGF-1 on LPS-induced airway inflammation *in vivo*. At 24 h after LPS challenge, mice showed significantly increased polymorphonuclear neutrophils (PMNs) in BALF, marked alveolar congestion and exudation, and increased inflammatory cell infiltration compared with that in normal control mice. Mice pretreated with IGF-1 directly into the airway 24 h before the establishment of the ALI model showed significantly reduced infiltration of inflammatory cells and a significantly lower number of PMNs and protein content in BALF than LPS treated mice (Figure 10). These data support the view that IGF-1 secretion by AMs is enhanced in response to AEC-derived TGF-β, which can serve as an important functional brake for the inflammatory response in alveoli during infection.

## Discussion

In this study, we showed that TGF-β produced by LPS-induced AECs stimulated the production of IGF-1 by AMs, and IGF-1 prevented the generation of pre-inflammatory signals in AECs and promoted the phagocytosis of apoptotic cells by AECs. IGF-1 is a polypeptide growth factor that plays a key role in regulating cell proliferation, differentiation, metabolism, and survival. It also demonstrated pleiotropy in some organs and was involved in the development of different disease states (25). For example, IGF-1 is closely related to the pathological processes of obesity, cardiovascular disease, central nervous system disease, tumor development, and other diseases. In addition, current research indicates that IGF-1 is associated with lung disease. IGF-1 is upregulated in patients with idiopathic and secondary pulmonary fibrosis (26). The level of IGF-1 mRNA is significantly increased in asthmatic patients (27). The serum IGF-1 level is decreased in chronic obstructive pulmonary disease patients in association with the severity of the disease (28). However, serum IGF-1 levels are elevated in ALI/ARDS patients (29). In the present study, we showed that IGF-1 levels were increased in the lung tissues of ALI model mice, and the IGF-1 in the airways of ALI model mice was derived from AMs. IGF-1 is produced in a variety of cells, including dental pulp stem cells, nucleus pulposus cells, macrophages, endothelial cells, and epithelial cells (17). Wang et al. recently reported that epidermal T cells can also produce IGF-1 (30). Han et al. showed that IGF-1 in Th2 type cytokine-stimulated mice is mainly derived from AMs, which is consistent with the present findings (31).

Stimulation of gastric vascular endothelial cells with nerve growth factor leads to the production of IGF-1 to prevent cells from damage caused by indomethacin (32). IL-15 promotes the repair of diabetic skin wounds by increasing the production of IGF-1 by epidermal T cells (30). In this study, we demonstrated that AEC-derived TGF-β induced IGF-1 production by AMs. TGF-β is a key factor for immune balance and tolerance that functions by preventing the expansion and activity of certain components of the immune system. It not only suppresses the function of adaptive immune cells such as T-cell DC cells, but also regulates the behavior of innate immune cells such as natural killer cells, macrophages, and neutrophils, which form a negative regulatory network in the immune system (33, 34).

TGF-β1 can both initiate the classic Smad signaling pathway and induce non-Smad signaling pathways such as the MAPK, Rho, and PI3K pathways (35, 36). In this study, we showed that TGF-β1 induced IGF-1 production in lung epithelial cells through the PI3K/Akt pathway. Wygrecka et al. demonstrated that TGF-β1 induces IGF-1 production in human lung fibroblasts through the PI3K/Akt pathway (37). The PI3K/Akt pathway also mediates the expression of type I collagen induced by TGF-β1 in retinal pigment epithelial cells and human mesangial cells (38). In vascular smooth muscle cells, TGF-β activates the PI3K/Akt pathway through Smad3 signaling, and p38 MAPK is the most important bridge connecting Smad3 and Akt (39). Both, the PI3K and Smad3 signaling pathways are involved in TGF-β1-induced cardiac fibroblast type I and type III collagen expression, and PI3K blockade reduces the phosphorylation of Smad3 initiated by TGF-β1. These findings indicate that the PI3K and Smad3 pathways may be integrated in the TGF-β1 signal (40).

Common signaling pathways between LPS and IGF-1 signals, such as PI3K/Akt and MAPK, may be involved in crosstalk between immune signals and endocrine signals. Exposure to IGF-1 prevents LPS-induced sickness behavior and the expression of pro-inflammatory factors in mice and rats (41, 42). IGF-1 prevents LPS-induced NOS2 expression in INS-1 cells by activating the PI3K/Akt signaling pathway (43). In buffalo granulocytes, IGF-1 downregulates LPS-induced expression of IL-1β, IL-6, and TNF-α, and delays LPS-induced phosphorylation of ERK1/2 (44). In this study, IGF-1 inhibited the increase of IL-1β, TNF-α, MCP-1 induced by LPS and the activation of p38 MAPK in AECs, whereas it had no effect on IL-6 production in AECs.

IGF-1 plays an important role in cell growth, migration, and differentiation, in addition to regulating the function of phagocytic cells. Exposure of mouse macrophages to IGF-1 significantly increases their phagocytic activity (45), whereas it does not affect the phagocytic function of rat macrophages (46). Jakn et al. showed that IGF-1 binds to IGF-1R on airway epithelial cells of non-professional phagocytic cells, which can promote the phagocytosis of microparticles by airway epithelial cells (31). In previous studies from our group, we showed that IGF-1 promotes the phagocytosis of fluorescent microspheres by alveolar epithelial cells (17). In this study, we demonstrated that IGF-1 promoted the phagocytosis of apoptotic cells by the alveolar epithelium. The phagocytosis of apoptotic cells by AECs is an important mechanism for suppressing airway inflammation. Our findings suggested that IGF-1 promoted the resolution of airway inflammation and accelerated the repair of inflammatory injury.

In this study, we showed that IGF-1 promoted the phagocytosis of apoptotic cells by lung epithelial cells by inducing the expression of PPARγ in lung epithelial cells. PPARγ and LXR are important transcription factors that regulate the expression of phagocytic receptors and bridge molecules to mediate phagocytosis (47). In this study, IGF-1 had no effect on the expression of LXR in AECs; therefore, related experiments focused on PPARγ.

Lee et al. showed that IGF-1 participates in the adipogenesis of mesenchymal stem cells by regulating the expression of PPARγ (48). In addition, IGF-1 induces the proliferation and invasion of UM cells by inhibiting Foxo3a (49). In hippocampal neural stem cells, IGF-1 upregulation activates the PI3K/Akt pathway, which upregulates the Brn4 transcription factor, thereby causing the differentiation of stem cells into neuronal phenotypes (50). NF-AT, CREB and other transcription factors are also activated by IGF-1 (51). These studies indicated that IGF-1 can activate multiple transcription factors to mediate its biological activity *in vivo*.

**Figure 11:**
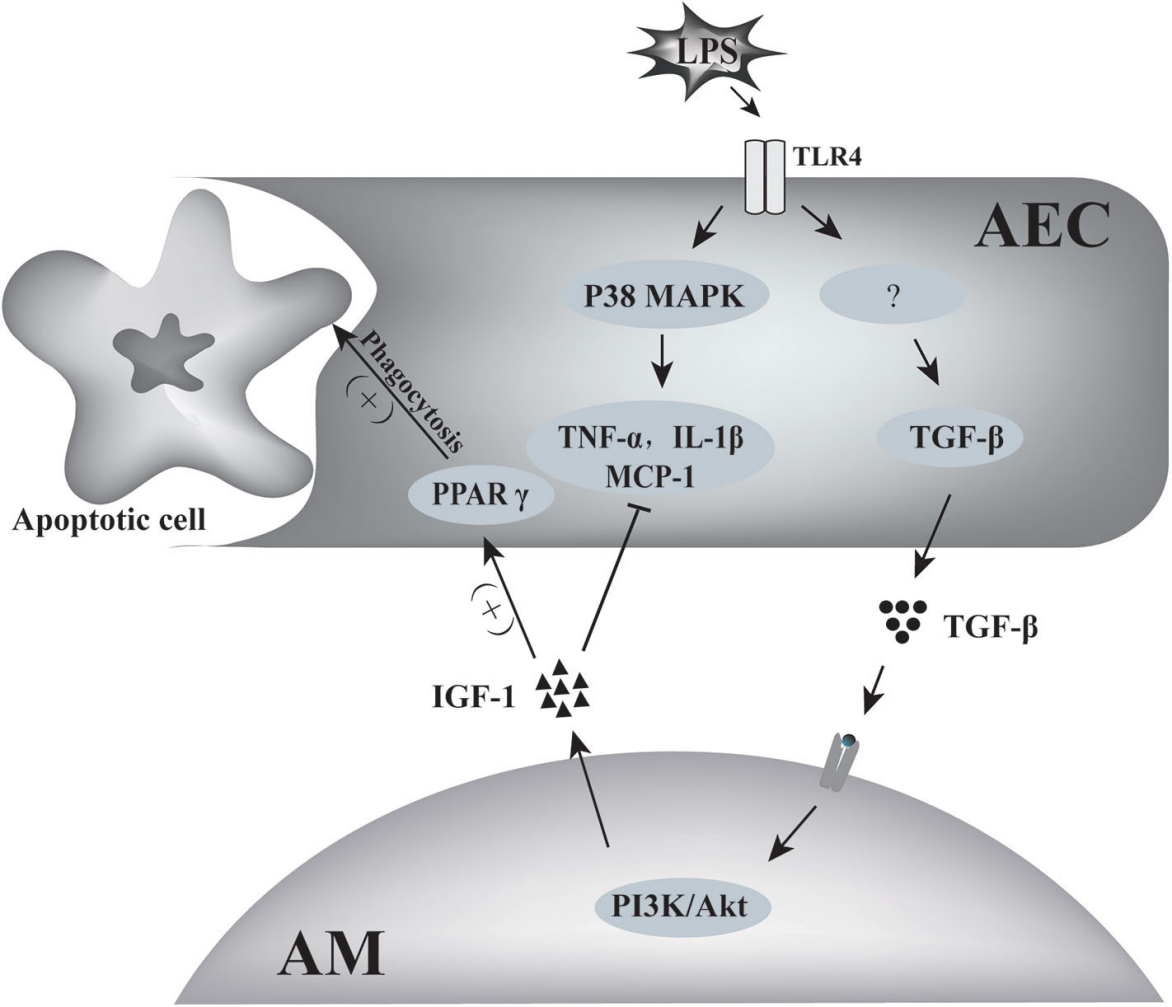
Schematic of the two-way communication between AMs and AECs in LPS-induced airway inflammation. LPS induced the production of inflammatory factors such as TNF-α, MCP-1, IL-1β by AECs through p38 MAPK signaling. AECs also produced TGF-β in response to LPS stimulation. TGF-β induced IGF-1 production by AMs through the PI3K/Akt signaling pathway. IGF-1 prevented LPS-induced p38 MAPK activation and MCP-1, TNF-α, and IL-1 β production in AECs, and it also promoted AEC phagocytosis of apoptotic cells through PPARγ.

In summary, the results of this study indicated that TGF-β1 derived from AECs activated AMs to secrete IGF-1 into the alveolar fluid in response to stimulation of the airway by inflammatory signals. This AM-derived IGF-1 attenuated the p38 MAPK inflammatory signal in AECs and promoted the phagocytosis of apoptotic cells by AECs. This two-way communication between AECs and AMs represents a well-tuned system for the regulation of the inflammatory response in alveoli (Figure 11).

## Data Availability Statement

The datasets presented in this study can be found in online repositories. The names of the repository/repositories and accession number(s) can be found in the article/supplementary material.

## Ethics Statement

The animal study was reviewed and approved by the Ethics Committee of Bengbu Medical College, Anhui, China.

## Author Contributions

MM and PG conceived and designed the experiments and performed the experiments. QY, JH, FW, XH, and SG conceived and designed the experiments, analyzed the data, and contributed reagents, materials, analysis tools. ZQ and CS analyzed the data, contributed reagents, materials, analysis tools, prepared figures and/or tables, and wrote the manuscript. All authors contributed to the article and approved the submitted version.

## Conflict of Interest

The authors declare that the research was conducted in the absence of any commercial or financial relationships that could be construed as a potential conflict of interest.
